# Correction: Visualizing cellular and tissue ultrastructure using Ten-fold Robust Expansion Microscopy (TREx)

**DOI:** 10.7554/eLife.85169

**Published:** 2022-11-29

**Authors:** Hugo GJ Damstra, Boaz Mohar, Mark Eddison, Anna Akhmanova, Lukas C Kapitein, Paul W Tillberg

**Keywords:** Human, Mouse

Damstra HGJ, Mohar B, Eddison M, Akhmanova A, Kapitein LC, Tillberg PW. 2022.7 Visualizing cellular and tissue ultrastructure using Ten-fold Robust Expansion8 Microscopy (TREx). *eLife* **11**:e73775. doi: 10.7554/eLife.73775.

Published 18 February 2022

In February 2022, we published our paper *Visualizing cellular and tissue ultrastructure using Ten-fold Robust Expansion Microscopy (TREx*), which introduced a simple and versatile approach for single-step ten-fold expansion of cells and tissue. We reported that the bisacrylamide concentration required for ten-fold expansion was different between labs in Ashburn and Utrecht, and suggested that the user test a range of bis concentrations to ensure ten-fold expansion. We have identified the source of this discrepancy to be a difference in the TEMED stock concentration between labs. As a result, the experiments in Figures 3E-G, 4 and 5 were performed with 1.5% TEMED and 90 ppm bisacrylamide, while the other experiments were performed with the reported TREx concentrations of 0.15% TEMED and 50 ppm bisacrylamide. After discovering this difference, we have replicated the experiments in Figures 3E-G, 4 and 5 with 0.15% TEMED and 50 ppm bis and obtained indistinguishable results. We have corrected the manuscript as follows:

**#1 (Discussion)**

Original text:

Therefore, we based our TREx recipe on recipe family D. The exact crosslinker concentration that produces 10-fold expanding gels was found to vary between labs (i.e., 50 µg/mL in Ashburn, VA, USA versus 90 µg/mL in Utrecht, the Netherlands, possibly due to differences in gelation chamber design), so we recommend that each lab test a range of crosslinker concentrations between 30 and 100 µg/mL using their choice of specimen, gelation chamber, and incubator.

Revised text:

Therefore, we based our TREx recipe on recipe family D. The exact crosslinker concentration that produces 10-fold expanding gels can vary due to differences in gelation chamber design, so we recommend that each lab test a range of crosslinker concentrations between 30–60 ppm using their choice of specimen, gelation chamber, and incubator.

**#2 (Materials and Methods)**

Original text:

TREx gelation solution contains 1.1 M sodium acrylate, 2.0 M acrylamide (AA), 50 µg/mL (for tissue slices and cultured cells prepared at Janelia), or 90 µg/mL (for cultured cells prepared at Utrecht University) N,N′-methylenebisacrylamide (bis), PBS (1×), 1.5 mg/mL APS, 1.5 mg/mL TEMED, and (optionally, for thick tissue slices) 15 µg/mL 4-hydroxy TEMPO (4HT, Sigma, 176141).

Revised text:

The TREx gelation solution contains 1.1 M sodium acrylate, 2.0 M acrylamide (AA), 50 ppm N,N’-methylenebisacrylamide (bis), PBS (1x), 1.5 ppt APS, 1.5 ppt TEMED, and (optionally, for thick tissue slices) 15 ppm 4-hydroxy TEMPO (4HT, Sigma, 176141).

**#3 (Materials and Methods)**

Original text:

For TREx, samples were treated with 100 µg/mL acryloyl-X SE (AcX) (Thermo Fisher, A20770) in PBS overnight at RT. TEMED and APS were added to monomer solution (1.5 mg/mL each) to produce gelation solution.

Revised text:

For TREx experiments shown in Figures 3E–G, 4 and 5, samples were treated with 100 μg/mL acryloyl-X SE (AcX) (Thermo Fisher, A20770) in PBS overnight at RT. In these experiments, TEMED and bis were used at a concentration of 15 ppt and 90 ppm, respectively.

